# An Amphipathic α-Helix Controls Multiple Roles of Brome Mosaic Virus Protein 1a in RNA Replication Complex Assembly and Function

**DOI:** 10.1371/journal.ppat.1000351

**Published:** 2009-03-27

**Authors:** Ling Liu, William M. Westler, Johan A. den Boon, Xiaofeng Wang, Arturo Diaz, H. Adam Steinberg, Paul Ahlquist

**Affiliations:** 1 Institute for Molecular Virology, University of Wisconsin-Madison, Madison, Wisconsin, United States of America; 2 National Magnetic Resonance Facility, University of Wisconsin-Madison, Madison, Wisconsin, United States of America; 3 Department of Biochemistry, University of Wisconsin-Madison, Madison, Wisconsin, United States of America; 4 Howard Hughes Medical Institute, University of Wisconsin-Madison, Madison, Wisconsin, United States of America; University of California San Francisco, United States of America

## Abstract

Brome mosaic virus (BMV) protein 1a has multiple key roles in viral RNA replication. 1a localizes to perinuclear endoplasmic reticulum (ER) membranes as a peripheral membrane protein, induces ER membrane invaginations in which RNA replication complexes form, and recruits and stabilizes BMV 2a polymerase (2a^Pol^) and RNA replication templates at these sites to establish active replication complexes. During replication, 1a provides RNA capping, NTPase and possibly RNA helicase functions. Here we identify in BMV 1a an amphipathic α-helix, helix A, and use NMR analysis to define its structure and propensity to insert in hydrophobic membrane-mimicking micelles. We show that helix A is essential for efficient 1a–ER membrane association and normal perinuclear ER localization, and that deletion or mutation of helix A abolishes RNA replication. Strikingly, mutations in helix A give rise to two dramatically opposite 1a function phenotypes, implying that helix A acts as a molecular switch regulating the intricate balance between separable 1a functions. One class of helix A deletions and amino acid substitutions markedly inhibits 1a–membrane association and abolishes ER membrane invagination, viral RNA template recruitment, and replication, but doubles the 1a-mediated increase in 2a^Pol^ accumulation. The second class of helix A mutations not only maintains efficient 1a–membrane association but also amplifies the number of 1a-induced membrane invaginations 5- to 8-fold and enhances viral RNA template recruitment, while failing to stimulate 2a^Pol^ accumulation. The results provide new insights into the pathways of RNA replication complex assembly and show that helix A is critical for assembly and function of the viral RNA replication complex, including its central role in targeting replication components and controlling modes of 1a action.

## Author Summary

Positive-strand RNA viruses (one-third of all virus genera) transfer their genetic material between host cells as RNA of mRNA polarity, which are translated into proteins immediately upon entry. One immediate function of these proteins is to establish RNA replication compartments on intracellular membranes to copy the incoming viral RNA. Although much is known about the viral protein and RNA components in such replication complexes, little is understood about how the multiple protein–membrane–RNA interactions required for replication complex assembly are regulated. To study this, we used a well-established model virus that encodes only two replication proteins: an RNA polymerase enzyme that copies the viral RNA and an assembly-coordinating protein that guides the rearrangement of intracellular membranes to form replication compartments and recruits the viral RNA template and polymerase to these sites. We identified a small helix in this guiding replication protein that is essential for efficient association with and rearrangement of the correct intracellular membrane type and for regulating a switch between at least two different functional states of the replication guide protein. Mutations in this small helix interfere with separable guide protein functions, revealing new insights into the sequential steps in positive-strand RNA virus RNA replication complex formation.

## Introduction

Positive-strand RNA viruses comprise over one-third of all virus genera and cause numerous diseases of humans, animals and plants [1]. Important human pathogens include hepatitis C virus (HCV), SARS coronavirus, Norwalk virus, West Nile virus, and the majority of common cold viruses, among others. Other positive-strand RNA viruses of animals, such as foot-and-mouth disease virus, and numerous plant viruses are of great veterinary and economic concern.

A universal feature of positive-strand RNA virus RNA replication is its close association with intracellular membranes. One or more viral nonstructural proteins target the viral replication complex to its preferred membrane type and often, if not always, induce membrane rearrangements. The responsible viral proteins can be true integral membrane proteins such as the flock house virus protein A that builds replication complexes on outer mitochondrial membranes [2] or HCV NS4B that targets HCV RNA replication to the endoplasmic reticulum (ER) membrane [3]. Alternatively, some viruses utilize peripheral membrane proteins such as the Semliki Forest virus nsP1 that locates to endosomal membranes [4] or HCV NS5A [5] and picornavirus 2 C [6], which associate with ER membranes.

Brome mosaic virus (BMV), a member of the alphavirus-like superfamily of human, animal, and plant viruses, is among the best-studied positive-strand RNA viruses for RNA replication. BMV has three genomic RNAs, RNA1, RNA2 and RNA3, and a subgenomic mRNA, RNA4. RNA1 and RNA2 encode nonstructural replicase proteins 1a and 2a polymerase (2a^Pol^), respectively, which are required for RNA replication. RNA3 and RNA4 encode the 3a movement protein and the coat protein, respectively, required for systemic spread in plants [7]. BMV RNA replication and encapsidation can be fully reconstituted in the yeast *Saccharomyces cerevisiae* by expressing the viral RNA replication and/or capsid proteins together with one or more genomic RNAs [8],[9],[10]. BMV replication in yeast duplicates the major features of replication in BMV's natural plant hosts, and the powerful techniques of yeast genetics and molecular biology have greatly facilitated the investigation of BMV replication and host-virus interactions [11],[12].

In plant cells and yeast, BMV RNA replication occurs on the perinuclear region of the ER [13]. The only viral component in the BMV RNA replication complex that localizes independently to the ER is replicase protein 1a [14], a multifunctional protein with an RNA capping domain in its N-terminal half and an NTPase/ RNA helicase-like domain in the C-terminal half [15],[16],[17]. The other viral RNA replication components, the RNA polymerase 2a^Pol^ and RNA templates, depend on 1a for their recruitment to the ER membrane and into the RNA replication process [14],[18],[19],[20],[21]. In close linkage with this recruitment, 1a dramatically increases the in vivo stability (but not the translation) of viral genomic RNA3 [22], and similarly increases the accumulation of the 2a^Pol^ protein [19].

When 1a associates with ER membranes, it induces the formation of membrane-bound spherular invaginations, that we will refer to as spherules [13]. By electron microscopy, the 50–70 nm diameter spherules are bounded by a single lipid bilayer continuous with the outer ER membrane and containing condensed or fibrillar material. The membrane bounding of this compartment is almost complete except for a narrow neck-like opening that retains a connection to the cytoplasm [13]. By electron microscopy, spherules in yeast cells that express only 1a are indistinguishable from spherules in yeast co-expressing 1a, low copy numbers of 2a^Pol^, and genomic RNA3, and that are actively replicating viral RNA [13]. Similar spherules are induced in association with RNA replication by many other positive-strand RNA viruses [12],[23].

The manner by which 1a interacts with ER membranes to induce these membrane invaginations, and the details of 1a's interactions with the other viral components remain poorly understood. We previously showed that BMV 1a has no trans-membrane domain(s) and resides fully on the cytoplasmic side of the ER membrane, but that amino acids 368–478 contain sequences important for ER membrane binding [24]. In this report we use NMR and other approaches to identify an amphipathic α-helix in this region, which is critically involved in 1a-membrane association, spherule induction and functional RNA replication complex assembly. The results also provide significant new insights into the pathways by which the RNA replication complex assembles and how different 1a functions are coordinated, revealing e.g. that 1a-induced membrane invagination and 1a-induced viral RNA protection are closely linked, while 1a interaction with and stimulation of BMV 2a^Pol^ accumulation does not require, and is in fact inhibited by, membrane rearrangements.

## Results

### A putative amphipathic α-helix in BMV 1a is sufficient for membrane association

**Figure 1:**
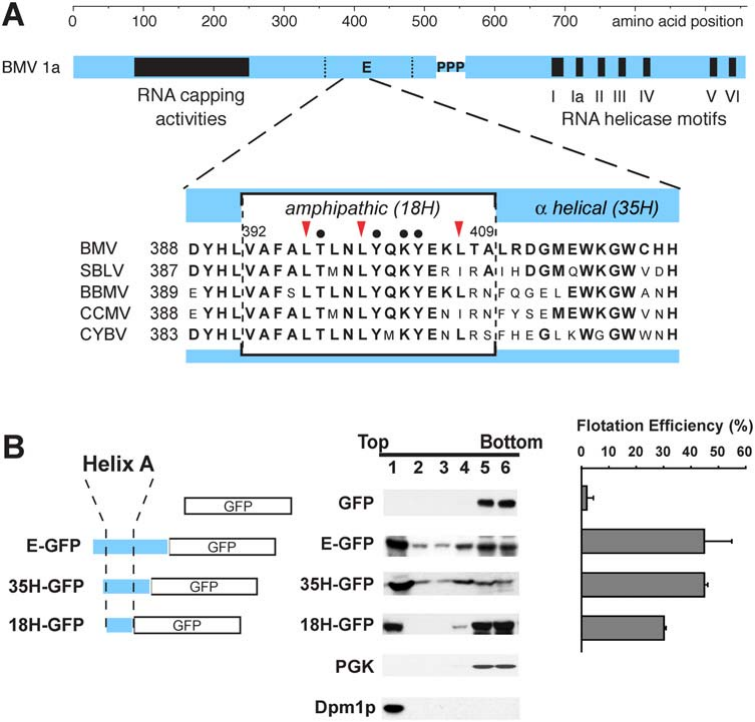
Evolutionarily conserved helix A is sufficient to direct membrane association of GFP. (A) A 35 amino acid region (35H) in membrane association domain E of BMV 1a is predicted to be α-helical and contains an amphipathic 18 amino acid core (18H, helix A). Alignments with the analogous sequences of other *Bromoviridae* members show a high level of absolute evolutionary conservation displayed in bold type. (SBLV, spring beauty latent virus; BBMV, broad bean mottle virus; CCMV, cowpea chlorotic mottle virus; CYBV, cassia yellow blotch virus). Red arrowheads indicate three leucines at BMV 1a positions 396, 400, and 407, and black dots indicate additional repeatedly referenced residues including threonine 397, tyrosines 401 and 404, and lysine 403. Amino acids (B) Distribution of GFP, E-GFP, 35H-GFP, 18H-GFP, PGK (cytosolic protein control), and Dpm1p (ER luminal protein control) in membrane flotation gradients. Representative western blots using anti GFP, anti-PGK, and anti-Dpm1p antisera are shown. Histograms show average flotation efficiencies based on three independent experiments. Flotation efficiency was calculated as the percentage of total protein in the gradient that segregated in the top two gradient fractions.

Previously, using membrane affinity and protease sensitivity assays, we showed that BMV 1a strongly localizes to the cytoplasmic face of the ER membrane despite lacking any detectable trans-membrane domain [24]. Membrane flotation assays of 1a deletion derivatives and GFP-fusion to truncated versions of 1a showed that a 105 amino acid (aa) region (aa 368–472, previously designated region E, Fig. 1A) plays a major role in 1a-ER membrane binding [24]. In this region, a stretch of 35 amino acids (aa 388–422) is predicted to be predominantly α-helical. Within this helical region, a putative amphipathic α-helix core peptide of 18 amino acids (aa 392–409) can be recognized, which we will refer to as “helix A”. One indication that helix A is likely important is that its amino acid sequence is evolutionarily highly conserved among the equivalent 1a replication proteins of other bromoviruses (Fig. 1A).

To test the functionality of helix A for membrane association, the 105, 35 and 18 aa regions described above were fused to the N-terminus of GFP to produce E-GFP, 35H-GFP, and 18H-GFP, respectively (Fig. 1B). Lysates of yeast cells expressing these fusion proteins were loaded under flotation gradients, which upon centrifugation were fractionated and analyzed by SDS PAGE and western blotting using anti-GFP antibodies. As a measure of membrane association, flotation efficiency was determined as the percentage of total GFP or 1a-GFP fusion protein in the gradient that was present in the top two fractions. In these assays, less than 3% of wild type cytosolic GFP floated to the top of the gradient with the membrane fraction. Fusing the 35 aa region to GFP greatly increased membrane association up to 45%, which was as efficient as membrane association directed by the full 105 aa E region fused to GFP. The smaller 18H-GFP fusion protein retained about 30% flotation efficiency (Fig. 1B). Thus, the 35 aa segment 388–422 accounts for essentially all of 1a's membrane association mediated by domain E, and the 18 aa helix A region retains most of this function and is sufficient to direct membrane association of GFP.

### NMR spectroscopy and mutational analysis confirm the α-helical and amphipathic nature of helix A

**Figure 2:**
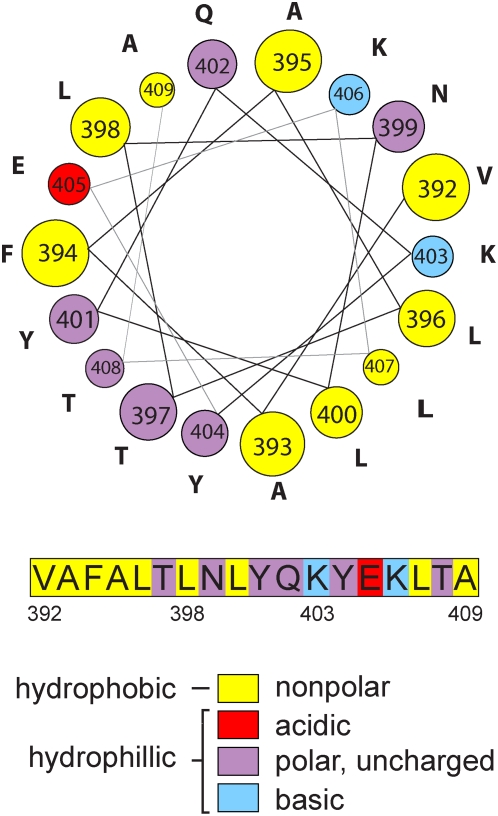
Linear and helical wheel projections of helix A. Color-coding indicates amino acid characteristics. Clustering of hydrophobic, non-polar residues on one face of the helix suggests an amphipathic configuration.

**Figure 3:**
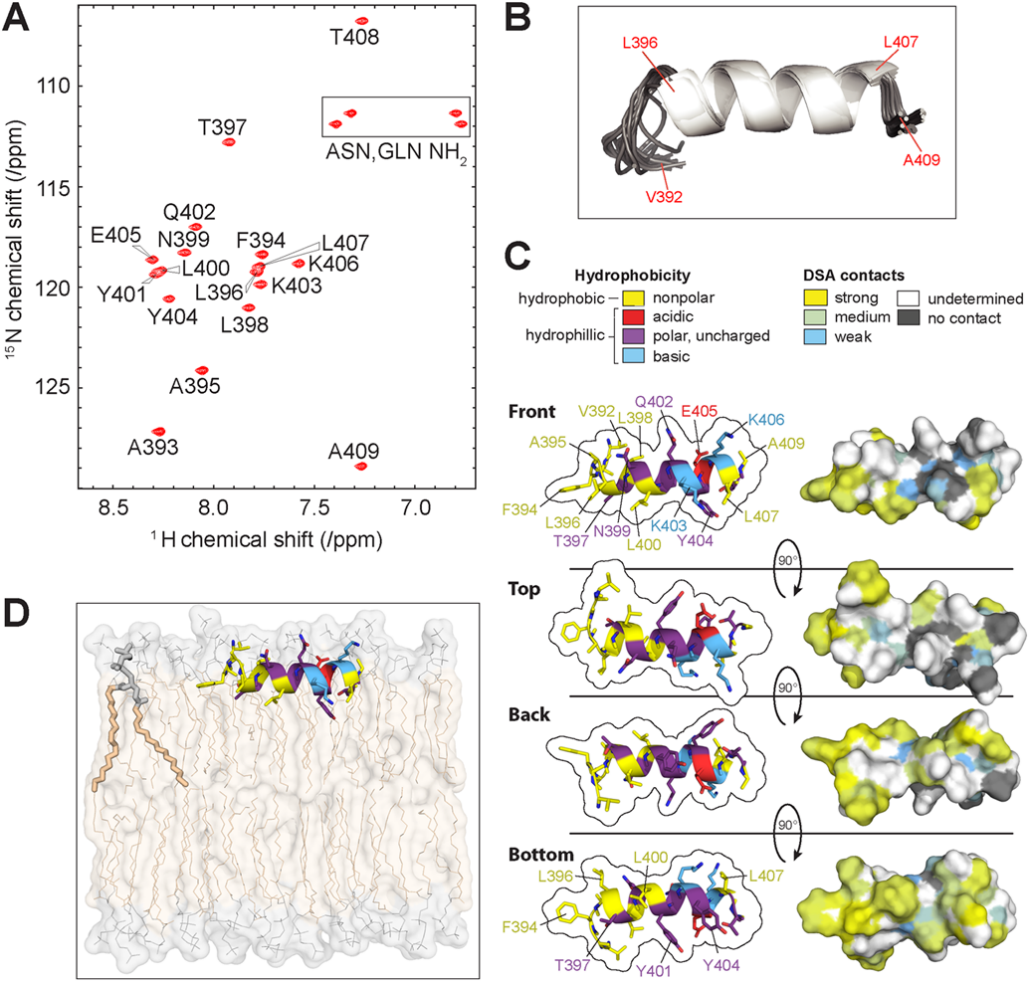
NMR structure of helix A on SDS micelles. (A) 1H{15N}-HSQC spectrum of BMV 1a helix A bound to 100 mM SDS micelles. Peaks arise from the amide moieties in the peptide. The assignments of peaks to particular amides are shown. Boxed peaks arise from the side chain amide protons of the Asn-399 and Gln-402 side chains and are unassigned. (B) Ensemble of 20 structures (backbone atoms only) determined for the peptide bound to an SDS micelle. The coloring represents the secondary structure as predicted on the basis of Ca and Cb chemical shifts [45]. White, helix; Gray, coil. (C) NMR-based three-dimensional structure of helix A in SDS micelles from four different viewpoints as indicated. (D) Artist's rendering of the topology of helix A at the interface between polar headgroups and fatty acid chains in a lipid bilayer, based on DSA contacts and other results discussed in the text. The “front” projection of helix A from panel C is shown. For comparison, the image of one of the glycerophospholipids is shown enhanced at the top left.

A helical wheel projection of the 18 aa helix A core region shows that it has the potential to form an amphipathic α-helical cylinder with one side (the right side in Fig. 2) having a cluster of hydrophobic, non-polar residues including three leucines (L396, L400, L407) and two nearby positive-charged lysines (K403, K406), and the other (left) side of the helix mostly hydrophilic and polar residues (Fig. 2, see also marked aa in Fig. 1A). To test these predictions, we used NMR to resolve the structure of an 18 aa peptide with the core sequence (aa 392–409) of helix A. NMR spectra of this peptide dissolved in water did not reveal a long term stable structure. However, upon including 100 mM SDS to provide lipid bilayer-mimicking micelles [25], the peptide showed NMR spectral changes consistent with a stable conformation (Fig. 3A). Based solely on ^13^C chemical shifts, NMR showed that aa 397–406 in the peptide had a >80% probability to be in a helical structure (Fig. 3B). To elucidate this further, the three dimensional structure of the peptide was calculated based on NOE distance constraints arising from spatial contact of hydrogen atoms observed to be closer than ∼5×. Additional dihedral angle constraints were derived from chemical shifts using the TALOS program [26]. The resulting structure (Fig. 3C) shows an α-helical conformation for aa 397–406, indicating that an amphipathic helix formed upon binding to the lipid membrane-mimicking SDS micelle. The constraints and overall quality of the structure are shown in Table 1.

**Table 1:** Statistics for the structure determination by NMR from PSVS [49].

**NOE-based constraints**	
Intraresidue (|i = j)	39
Sequential (|i−j| = 1)	45
medium range [1<|i−j|<5]	37
long range [|i−j|≥5]	3
Total	124
Dihedral angle constraints from Talos [26]	24
Number of structures	20
**Ramachandran data**	
Most favored regions	89.6%
Additionally allowed regions	10.4%
Generously allowed regions	0.0%
Disallowed regions	0.0%
**RMSD from experimental constraints**	
All backbone atoms	0.2 Å
All heavy atoms	0.9 Å

Table 2 shows that 65% of the observed NMR signals were assigned to specific atoms in the peptide. Of these assigned signals, 80% were affected by the addition of 16-doxyl stearic acid (DSA), a paramagnetic molecule whose presence in SDS micelles causes nearby atoms' NMR signals to broaden and lose intensity, thus serving as an internal probe for the extent to which atoms on the surface of a labeled structure are immersed in the micelles [27]. In parallel with the distribution of hydrophobic amino acid residues (Fig. 2 and Fig. 3C), the N-terminal half of the peptide had a larger percentage of assigned atoms that showed DSA contact than the C-terminal half, i.e. 91% vs. 69%, respectively (Fig. 3C and Table 2).

**Table 2:** Titration results for BMV-1a helix A bound to SDS micelles with 16-DSA.

	% of total peaks		
	aa 392–409	aa 392–400	aa 401–409
Assigned peaks	65	66	64
In contact with micelle^a^	80	91	69
No contact with micelle^b^	20	9	31

a 50% signal intensity decrease with the addition of less than 3.2 mM DSA.

b Required more than 3.2 mM DSA to lose 50% intensity.

**Figure 4:**
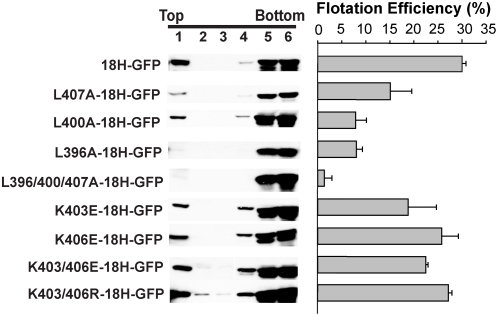
Membrane flotation analysis of helix A-GFP fusion protein and its mutant derivatives. Representative western blots using anti GFP antiserum are shown. Histograms on the right show average flotation efficiencies based on three independent experiments, calculated as in Figure 1.

Since the structure and DSA results implied that L396, L400, and L407 were positioned in the face of helix A most deeply immersed into the bilayer-mimicking micelle (Fig. 3C, bottom view, and 3D), we tested the importance of these three leucines for helix A-mediated membrane association. We introduced L to A mutations in the 18H-GFP fusion protein expression plasmid and tested their effects on membrane flotation efficiency. As shown in Fig. 4, the wt18H-GFP again had 30–35% flotation efficiency, while single L to A mutations reduced this to ∼7–15%. Of the three leucines, mutating the more N-proximal L396 and L400 more severely reduced membrane association than mutating L407, which paralleled the stronger micelle contact of the N-terminal half of the peptide (Fig. 3C and Table 2). These results might also explain in part the tolerance for an isoleucine at the 407-equivalent position in other bromovirus replicase proteins (Fig. 1A). A fusion protein with a combination of all three L to A mutations had near background level flotation, implying a complete loss of function of helix A in targeting cytosolic GFP to membranes. In contrast, K to E mutations reversing the charge of lysines 403 and 406 (the only basic residues in the 18 aa helix A core) showed K403E to only marginally decrease the flotation efficiency of 18H-GFP, while K406E had no significant effect (Fig. 4), consistent with the NMR observation that these amino acids have weak and no lipid contact, respectively (Fig. 3C). A double K to R mutation designed to retain the positive charge at these amino acid positions did not affect membrane association at all (Fig. 4), suggesting that K403 might contribute to membrane association via its positive charge, perhaps by neutralizing negatively charged lipid head groups. Overall, as mutations that change the leucine-rich non-polar face of the helix have more detrimental effects on membrane association than other amino acid substitutions, the results were consistent with the NMR-based structure of helix A and show that amphipathic helix A has a key role in membrane targeting.

### Helix A is required for efficient membrane association of full-length 1a

**Figure 5:**
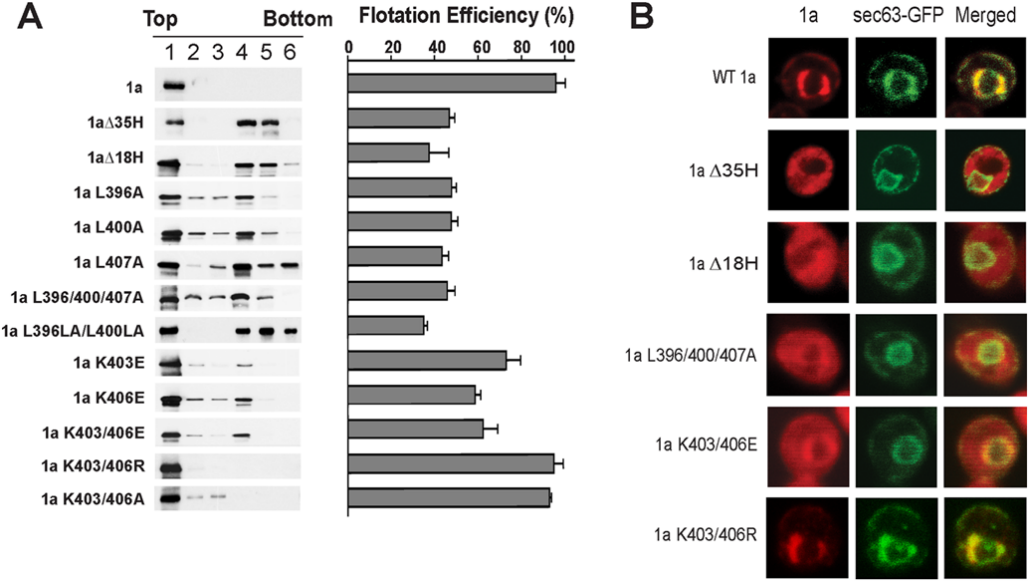
Helix A and specific non-polar (L) and polar residues (K) in it are required for efficient 1a membrane binding and ER targeting. (A) Distribution of wt 1a or 1a helix A mutants in membrane flotation gradients. Representative western blots using anti-1a antiserum are shown on the left, the histogram on the right shows average flotation efficiencies based on three independent experiments, calculated as in Figure 1. The levels of commonly observed less-than-full-length 1a-derived degradation products (whose separation from 1a depends on the particular gel composition and run time used) were not included in the calculations. (B) Fluorescence microscopy images of cells expressing wt 1a or 1a helix mutants and Sec63-GFP as an ER marker.

To extend the results from helix A-GFP fusion proteins, the contribution of helix A to membrane association of full-length 1a was assessed using biochemical and cell imaging approaches. By membrane flotation gradient analyses, the flotation efficiency of wt 1a was ∼ 96% (Fig. 5A), confirming 1a's previously established high affinity for membranes [24]. Deleting the 35 aa or 18 aa helices reduced 1a-membrane association by over two-fold (Fig. 5A). The three L to A mutations, either as single mutations or as a triple combination, similarly reduced the flotation efficiency of full length 1a to ∼45%. Single alanine insertions immediately downstream from L396 and L400 reduced flotation efficiency to levels similar to full helix A deletions (Fig. 5A), confirming the importance of correct spacing to maintain the amphipathic characteristics of helix A. The importance of the charged lysines at positions 403 and 406 at the hydrophilic face of helix A was assessed using alanine or arginine substitutions. Single position substitution mutants and double mutants K403/406A and K403/406R maintained full flotation efficiency (Fig. 5A, single mutations not shown). 1a mutants K403E, K406E, and double mutant K403/406E retained intermediate flotation efficiencies showing that although the positive charge at these positions is not required, reversing it to a negative charge destabilizes membrane association (Fig. 5A). The K403/406E single and double mutations showed a somewhat greater inhibition of membrane association in the context of full length 1a (∼63% for the double mutant in Fig. 5) than in the context of the 18 aa helix fused to GFP (∼77%, Fig. 4), suggesting the possibility that residues outside of the 18 aa helix core might cooperatively influence membrane association.

Since none of the deletions and mutations completely abolished 1a-membrane association, we used confocal immunofluorescence microscopy to compare the sub-cellular localization of the 1a mutants with that of wt 1a (Fig. 5B). Wildtype 1a localized predominantly to the perinuclear ER membrane, co-localizing almost completely with the distribution of ER marker Sec63p. In contrast, the 1a protein mutants that lacked either the 35aa or 18aa helices no longer co-localized with Sec63p and displayed a mostly diffuse cytoplasmic localization (Fig. 5B). Confocal fluorescence images showed similar staining throughout the cytoplasm for 1a triple mutant L396/400/407A and the K403/406E double mutants, although in these cases a minority of 1a retained ER association. By contrast, the K403/406R mutant co-localized with Sec63p throughout, as for wt 1a (Fig. 5B).

Combined, the flotation and confocal results demonstrate that 1a has both helix A-dependent and -independent modes of membrane association, but that helix A is crucial for efficient membrane association and normal 1a localization to perinuclear ER membranes. While other aa such as the positively charged lysines contribute, the leucines on the hydrophobic side of helix A are the most important residues for effective association of 1a with ER membranes.

### Helix A determines the type and ultrastructure of 1a-induced ER membrane rearrangements

**Figure 6:**
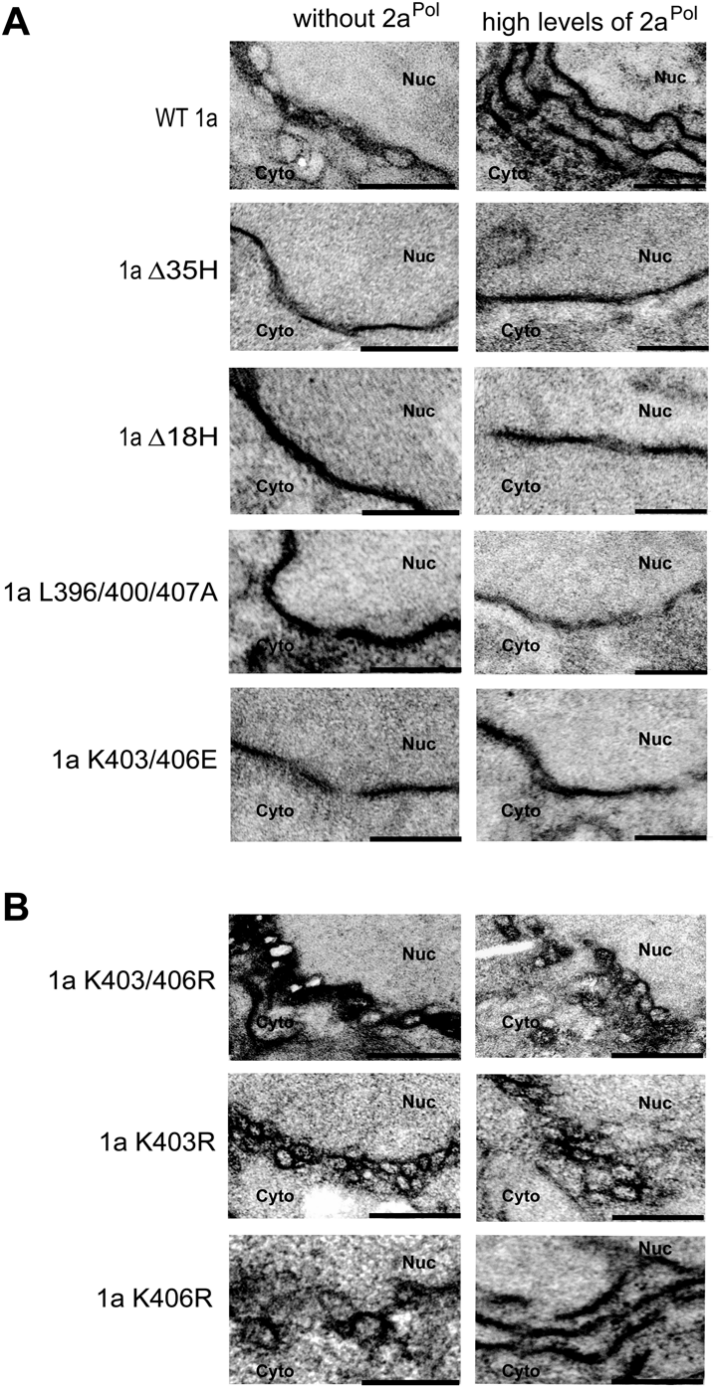
Helix A and specific non-polar (L) and polar residues (K) in it are required for 1a-induced ER membrane rearrangements. (A) EM images of membrane spherules or layers or the absence thereof in yeast cells expressing wt 1a or 1a helix A mutants when expressed alone (left) or when co-expressed with high levels of 2a^Pol^ (right). (B) 1a double mutant K403/406R produces much more numerous, somewhat smaller spherules than wt 1a, and this phenotype is maintained in single mutant 1a K403R, but not 1aK406R. Nuc, nucleus; Cyto, cytoplasm. (Scale bars, 200 nm.)

We previously showed that, in the absence of 2a^Pol^ or other viral components, 1a targets itself to perinuclear ER membranes and induces spherular invaginations that by EM are indistinguishable from those that replicate BMV RNA when 1a is expressed together with low 2a^Pol^ levels expressed from the yeast *ADH1* promoter [13]. Examples of such spherules are shown in Fig. 6, top left panel. In contrast, 1a plus high 2a^Pol^ levels expressed by the strong yeast *GAL1* promoter shift the predominant viral-induced membrane rearrangements from spherules to large, karmellae-like, multilayer stacks of double membrane layers surrounding the nucleus (Fig. 6, top right panel). Although dramatically different in organization, such membrane layers support BMV RNA replication as efficiently as spherules [28].

Fig. 6 shows that deleting helix A (1aΔ35H, 1aΔ18H) abrogated 1a's ability to induce either type of ER membrane rearrangement. Likewise, mutating the hydrophobic face of helix A in triple mutant 1a L396/400/407A or reversing the positive charge of the two lysines in double mutant 1a K403/406E abolished 1a's ability to form either membrane rearrangement, whether expressed alone or together with *GAL1*-promoter -driven 2a^Pol^ (Fig. 6A). Arginine substitution of the single negatively charged amino acid E405 maintained a wt phenotype, although alanine substitution at this position resulted in a >30-fold reduction in the number of spherules, showing the importance of a charged, hydrophilic amino acid at this position (data not shown).

In surprising contrast, double mutant 1a K403/406R showed an entirely different phenotype. This mutant, which as described earlier maintained full flotation efficiency (Fig. 5A), was revealed by EM analysis to form dramatically more, and somewhat smaller, membrane-bound spherules than wt 1a (Fig. 6B). To specify which of the two amino acid changes contributed to this phenotype, single mutants 1a K403R and 1a K406R were generated and expressed in yeast cells. In keeping with the DSA/membrane interaction of K403 but not K406 (Fig. 3C), Fig. 6B shows that 1a K403R maintained this mutant phenotype while 1aK406R induced spherules with the frequency and size of wt 1a. Moreover, 1a K403R induced high frequency, smaller spherules even in the presence of high levels of *GAL1*-promoter-driven 2a^Pol^ expression (Fig. 6B), conditions under which wt 1a preferentially induces ER membrane layers rather than spherules (Fig. 6A). These results show both that 1a-ER membrane association through helix A is crucial for 1a-induced membrane rearrangements, and that additional characteristics of helix A have important roles in determining the type of membrane rearrangement and the extent of membrane curvature.

**Figure 7:**
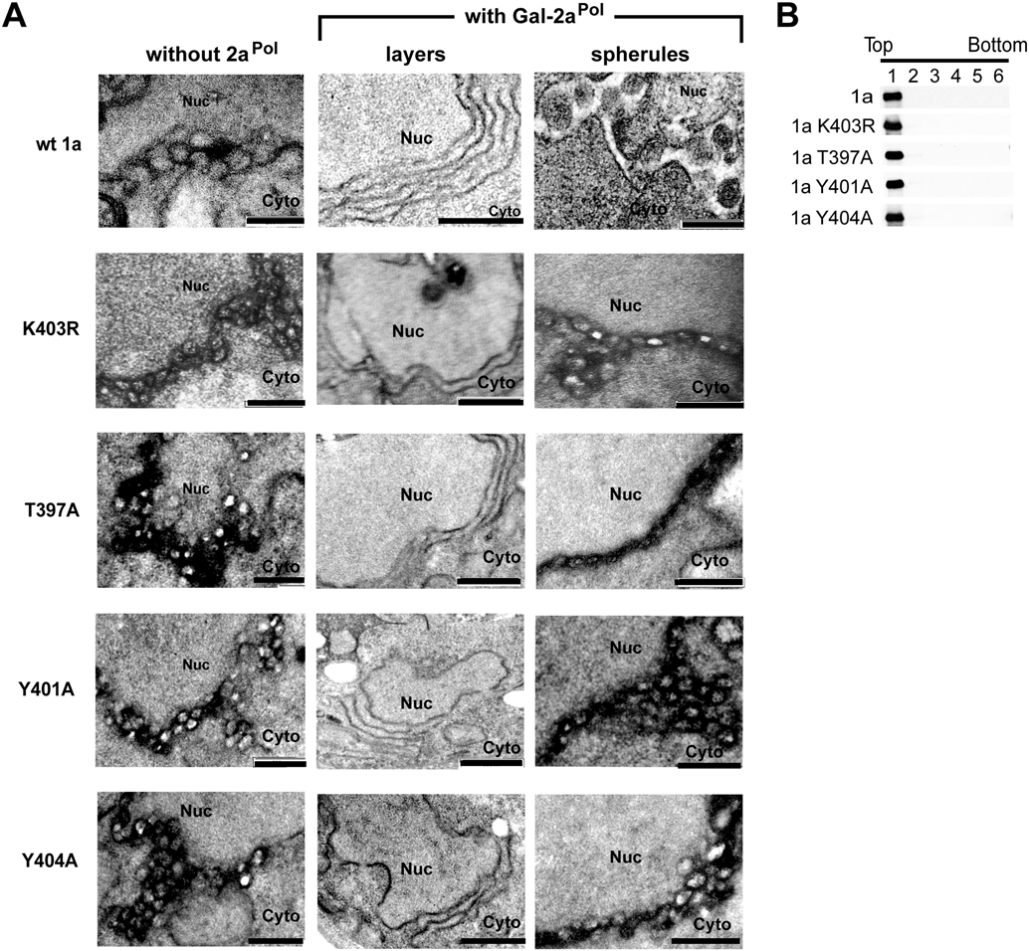
Class II mutations in 1a helix A induce hyper-abundant membrane invaginations. (A) EM images of membrane spherules in yeast cells expressing wt 1a or helix A 1a mutants alone (left), or membrane layers or spherules in cells co-expressing high levels of 2a^Pol^ (center and right, respectively). Nuc, nucleus; Cyto, cytoplasm. (Scale bars, 200 nm in top and bottom panels, 500 nm in center panels.) (B) Distributions of wt 1a and Class II 1a helix A mutants in membrane flotation gradients.

Hereafter, we will refer to helix A mutants that have lost all membrane-rearranging capacity, like triple mutant L396/400/407A, as Class I mutants, and to mutants with the hyper- abundant, smaller spherule phenotype, like K403R, as Class II mutants. To evaluate the possible role of other helix A amino acids in Class I or Class II phenotypes, we first made alanine substitutions at the other residues besides L396/400/407 in the major membrane interacting face of helix A, i.e., F394, T397, Y401, Y404 and T408 (Fig. 3C, bottom view; see also Fig. 2). Strikingly, EM analysis showed that 1a T397A, 1a Y401A and 1a Y404A all were Class II mutants, inducing a plethora of small spherules like 1a K403R (Fig. 7A). Flotation analyses showed that all four of these Class II mutants also maintained wt 1a levels of membrane association (Fig. 7B). The F394A substitution, positioned on the same side of helix A as the above Class II mutants but at the N-terminal end of helix A (Fig. 2 and Fig. 3C), had a partial Class II phenotype of producing spherules at normal frequency but slightly smaller diameter than wt 1a. An alanine substitution at K403 resulted in a similar phenotype. By contrast, spherules of wt frequency and size were produced by 1a T408A, at the C-terminal end of helix A, by 1a bearing alanine substitutions at residues on the upper face of helix A (Fig. 3C), i.e., V392, L398, N399, and Q402. and by 1a A395S (results not shown).

To more accurately and precisely describe the Class II mutant phenotypes, we measured the abundance and diameter of spherules in the subset of cells that were sectioned through their nuclei among a total of 200 cells for each mutant. As shown in Table 3, spherule abundance in yeast cells expressing Class II mutants was 5- to 7- fold higher than in cells expressing wt 1a. Moreover, the average spherule diameter in cells expressing wt 1a was ∼66 nm, but was only ∼40–55 nm in cells expressing Class II mutants.

**Table 3:** Effects of two classes of helix A mutations on 1a-induced ER membrane rearrangements, BMV RNA3 accumulation, RNA replication, 1a and 2a^Pol^ accumulation, and localization.

1a or 1a mutant	frequency of spherules^a^	with GAL-2a^Pol^		average spherule diameter^c^ (nm)	RNA3 accumulation^d^	RNA3 replication	1a accumulation	2a^Pol^ accumulation	1a and 2a^Pol^ localization
		frequency of spherules^a^	frequency of cells with layers^b^						
wt 1a	100%	100%	100%	66±16	100%	100%	100%	100%	ER
1aΔ35H	0%	0%	0%	-	2%	0%	100%	210%	ER and cytoplasm
1aΔ18H	0%	0%	0%	-	2%	0%	100%	221%	ER and cytoplasm
1a L396/400/407A	0%	0%	0%	-	5%	0%	100%	219%	ER and cytoplasm
1a K403/406E	0%	0%	0%	-	6%	0%	100%	215%	ER and cytoplasm
1a T397A	509%	697%	28%	41±8	204%	23%	100%	74%	ER
1a Y401A	475%	698%	29%	47±8	203%	2%	100%	42%	ER
1a Y404A	645%	578%	17%	49±8	167%	2%	100%	56%	ER
1a K403R	581%	807%	6%	55±10	214%	2%	100%	45%	ER

a (average number of spherules in yeast cells with a clear nucleus in the plane of section among a total of two hundred yeast cells sectioned / average number of spherules in wt 1a expressing yeast cells with a clear nucleus in the plane of section among a total of two hundred yeast cells sectioned) ×100%.

b (average number of yeast cells with a clear nucleus and layers in the plane of section among a total of two hundred yeast cells sectioned / average number of wt 1a expressing yeast cells with a clear nucleus and layers in the plane of section among a total of two hundred yeast cells sectioned) ×100%.

c average of independently measured diameters of 50 spherules±standard deviation.

d [(RNA3 signal - RNA signal without 1a)/(RNA3 signal with wt 1a – RNA signal without 1a)] ×100%.

As mentioned earlier, when wt 1a and high levels of 2a^Pol^ are co-expressed, only 15–25% of cell sections with BMV-induced, perinuclear membrane rearrangements show spherules, while 75–85% bear double-membrane layers that support efficient RNA replication [28]. Even under such conditions of high 2a^Pol^ expression, the four Class II mutants induced ∼6- to 8-fold more spherules than wt 1a and reduced the frequency of cells with double membrane layers by >3- to 10-fold (Table 3). Thus, helix A mutations not only alter 1a's intrinsic functions for ER membrane rearrangement, but also the ability of 2a^Pol^ to modulate the type of 1a-induced ER membrane rearrangements.

### 1a-stimulation of 2a^Pol^ accumulation does not require formation of new membrane compartments

**Figure 8:**
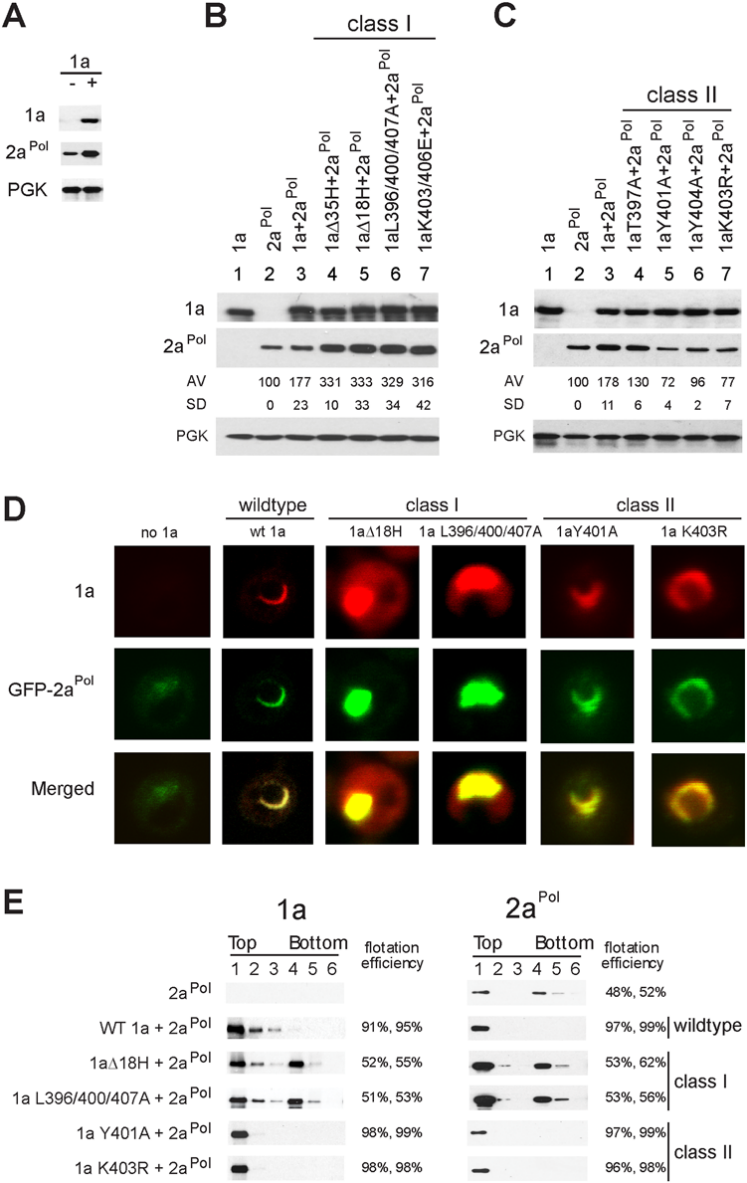
Class I and Class II mutations in 1a helix A have opposite effects on 2a^Pol^ accumulation. (A) 2a^Pol^ protein expression levels in yeast cells expressing either 2a^Pol^ alone or co-expressing 2a^Pol^ and wt 1a. Levels of cytosolic protein PGK were measured as a loading control. (B) 1a, 2a^Pol^, and PGK protein expression levels in yeast cells expressing either 1a alone, 2a^Pol^ alone, 2a^Pol^ and wt 1a, or 2a^Pol^ and Class I 1a mutant derivatives that abolished 1a-induced ER membrane rearrangement. (C) 1a, 2a^Pol^, and PGK protein expression levels in yeast cells expressing either 1a alone, 2a^Pol^ alone, 2a^Pol^ and wt 1a, or 2a^Pol^ and Class II 1a mutant derivatives that induced more but smaller spherules. (D) Fluorescence microscopy images of cells expressing GFP-2a^Pol^ alone or co-expressed with wt 1a or the indicated 1a mutants. (E) Distributions of wt 1a, 1a mutants and 2a^Pol^ in membrane flotation gradient analyses of lysates from yeast cells expressing 2a^Pol^ alone or co-expressing 2a^Pol^ and wt 1a or the indicated 1a mutants. Representative flotation efficiencies from two independent experiments, calculated as in Figure 1, are shown.

In addition to mediating its own membrane association, wt 1a also recruits 2a^Pol^ to the RNA replication complex, mediated at least in part by a direct interaction between 1a's C-terminus and the N-terminus of 2a^Pol^ [19],[29]. In conjunction with such recruitment in this and previous studies [19], co-expressing wt 1a increased 2a^Pol^ accumulation by approximately two-fold (Fig. 8A). Accordingly, we measured 2a^Pol^ accumulation in the presence of the various 1a mutants to determine to what extent this 1a function depended on sequences in helix A. All mutant 1a proteins accumulated to levels similar to wt 1a, but Class I and Class II mutants showed directly opposite effects on 2a^Pol^ accumulation (Fig. 8B and 8C). Class I 1a mutants that lack the ability to induce ER invaginations not only retained the ability to stimulate 2a^Pol^ accumulation, but did so to nearly double the level of wt 1a (Fig. 8B). In contrast, Class II 1a mutants that form more numerous, smaller spherules, lost the ability to stimulate 2a^Pol^ levels over those in cells expressing 2a^Pol^ alone (Fig. 8C). Thus, 1a-mediated stimulation of 2a^Pol^ accumulation was inversely correlated with the capacity of 1a to induce ER membrane invaginations.

The localization of wt 1a and selected representatives of the Class I and II 1a derivatives in cells co-expressing 2a^Pol^ is shown in Fig. 8D (see also Fig. 5B for localization of the 1a derivatives without 2a^Pol^). For each 1a mutant class, similar results were obtained with all members, and representative results are shown in Fig. 8D. In these studies we used a replication-competent GFP-2a^Pol^ fusion protein to allow direct fluorescence microscopy detection rather than immunofluorescence, which is often compromised by low 2a^Pol^ detection sensitivity [19].

As seen previously [19], GFP-2a^Pol^ fluorescence in the absence of 1a was mostly faint and diffusely cytoplasmic with a few punctate dots. When co-expressed with wt 1a, GFP-2a^Pol^ co-localized with 1a in typical partial to almost complete ring-like perinuclear ER structures (Fig. 8D), consistent with prior observations [19]. Although Class II 1a mutants failed to significantly stimulate 2a^Pol^ accumulation, the GFP-2a^Pol^ that accumulated in cells expressing Class II mutants co-localized with the mutant 1a in perinuclear rings similar to wt 1a (Fig. 8D, right two columns). By contrast, in the presence of the reduced membrane affinity Class I 1a mutants, GFP-2a^Pol^ accumulated in large cytoplasmic clusters also containing a significant fraction of the mutant 1a, while the remaining 1a was distributed diffusely over the cytoplasm (Fig. 8D, third and fourth columns), as when these Class I mutants were expressed without 2a^Pol^ (Fig. 5B).

As another assessment of membrane association, flotation efficiency of 1a or its helix A mutants remained unaffected when co-expressed with 2a^Pol^ (compare Fig. 8E with Fig. 5A). In the presence of wt 1a, 2a^Pol^ accumulation was stimulated and essentially all 2a^Pol^ became membrane-associated (Fig. 8E). Likewise, 2a^Pol^ was recruited to membranes by the Class II 1a mutants with ∼98% efficiency, but without any increased accumulation (Fig. 8E). When co-expressed with any Class I 1a mutants, the efficiency of 2a^Pol^ flotation with membranes was only 50–60% (Fig. 8E), slightly higher than without 1a and similar to the reduced membrane-association of Class I mutant 1a proteins themselves, with or without 2a^Pol^ (Fig. 8E and 5A).

### RNA3 requires 1a-induced membrane invaginations to acquire a membrane-associated, nuclease-resistant state

**Figure 9:**
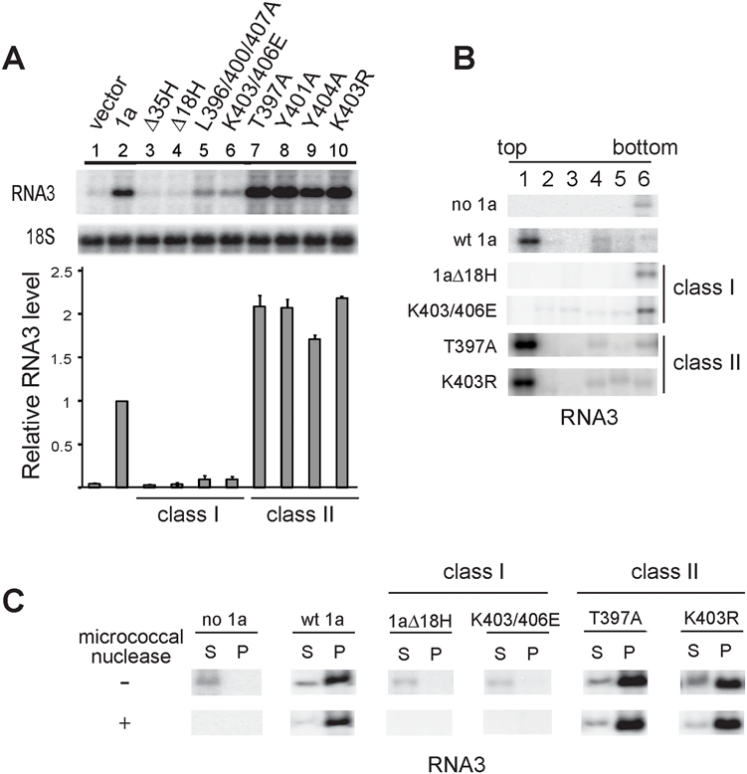
Class I and Class II mutations in 1a helix A have opposite effects on recruiting genomic RNA3 to a membrane-associated, nuclease-resistant state. (A) RNA3 levels in yeast cells expressing RNA3 either alone, with wt 1a, or with 1a bearing Class I or Class II mutations in helix A. 18S rRNA was measured as a loading control. (B) Distribution of RNA3 in membrane flotation gradients in yeast cells expressing either RNA3 alone or with wt 1a or Class I or Class II 1a helix A mutants. (C) Distribution of RNA3 in supernatant (S) and pellet (P) fractions of lysates incubated with or without micrococcal nuclease from yeast cells expressing RNA3 alone or with wt 1a or Class I or Class II 1a helix A mutants.

In yeast cells, the half-life of RNA3 increases from 5–10 min in the absence of 1a to more than 3 hours in the presence of 1a, which is reflected in a marked increase in RNA3 accumulation [22]. Accordingly, as shown in Fig. 9A, lanes 1 and 2, *GAL1* promoter-driven wt 1a increased RNA3 accumulation ∼20-fold. Strikingly, the effects of the Class I and Class II mutations on 1a stimulation of RNA3 accumulation were opposite to each other and opposite to the effects of each mutant on 2a^Pol^. Co-expressing class II 1a mutants stimulated RNA3 accumulation ∼40-fold, or double the stimulation by wt 1a (Fig. 9A), in parallel with the increased frequency of spherule formation by these mutants (Table 3). In contrast, Class I 1a mutants showed no ability to stimulate RNA3 accumulation, so that RNA3 levels in cells expressing Class I 1a mutants were similar to those in cells lacking 1a (Fig. 9A).

Wild type 1a recruits RNA3 into a membrane-associated, nuclease-resistant state [13]. To define the state of RNA3 in the presence of the Class I and Class II 1a mutants, we assayed RNA3's membrane flotation efficiency, sedimentation, and nuclease sensitivity when co-expressed with these mutants (Fig. 9B and 9C). Without wt 1a, RNA3 remained at the bottom of flotation gradients, indicative of a complete lack of membrane-association. In sedimentation assays, RNA3 from cells lacking 1a was mainly detected in the membrane-depleted supernatant and readily degraded with micrococcal nuclease (Fig. 9C). In the presence of wt 1a or its Class II mutants, at least 80% of RNA3 segregated with the membrane fraction in the top gradient fractions or the membrane-enriched pellet fraction in sedimentation assays, and became highly nuclease-resistant, while Class I mutants failed to induce RNA 3 membrane association or nuclease resistance (Fig 9B and 9C). Thus, the loss or enhancement by Class I or II 1a helix A mutants of wt 1a's ability to stimulate RNA3 accumulation in vivo was closely linked with RNA3's acquisition of a membrane-associated, nuclease-resistant state, and with the capacity of each 1a mutant's ability to induce membrane invaginations.

### Helix A mutations abolish BMV RNA replication in yeast and a natural plant host

In cells expressing 1a and 2a^Pol^, RNA3 transcripts are recruited into 1a- and 2a^Pol^-containing replication complexes to serve as templates for synthesis of negative-strand RNA3, which in turn becomes the template for synthesis of progeny positive-strand RNA3 and subgenomic positive-strand RNA4 [8],[21]. Since the Class I and Class II mutants in 1a helix A show opposite effects on 1a-associated intracellular localization, ER membrane rearrangements and stimulation of 2a^Pol^ and RNA3 accumulation (Table 3) we compared how these mutants affect BMV RNA replication in yeast and in a natural plant host of BMV, barley.

**Figure 10:**
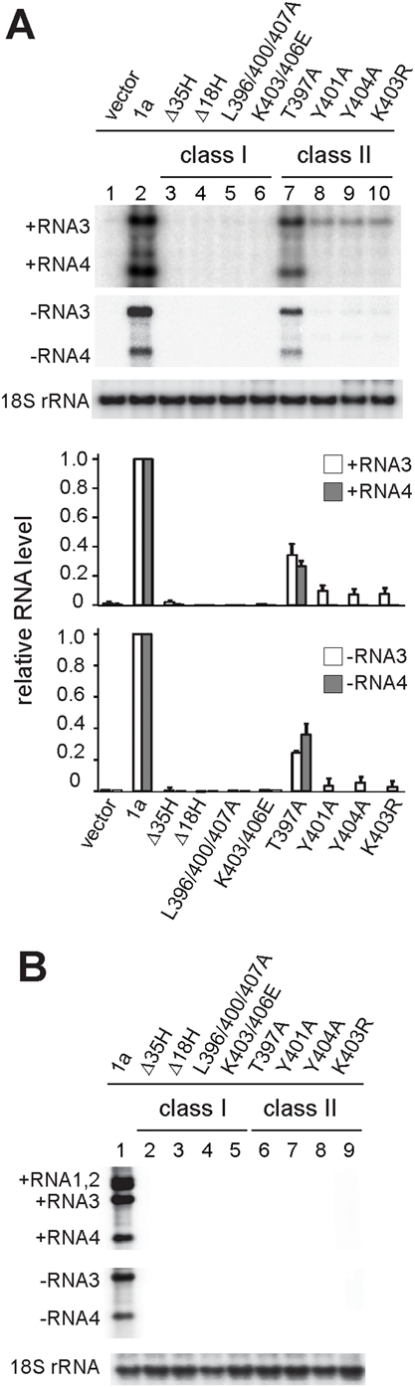
1a helix A mutations abolish BMV RNA replication in yeast cells and barley. (A) Analysis of positive and minus strand RNA3 and RNA4 from yeast cells expressing 2a^Pol^, RNA3 and either wt 1a or Class I or Class II 1a helix mutants. (B) Viral RNA levels in barley leaves 9 days post-inoculation with *in vitro* transcribed wt RNA1 or helix A mutant RNA1 and RNA2 and RNA3. 18S rRNA was measured as a loading control in (A) and (B).

Yeast cells expressing wt 1a, 2a^Pol^ and RNA3 supported efficient viral RNA replication (Fig. 10A). In contrast, in cells expressing Class I 1a mutants, 2a^Pol^ and RNA3, only weak RNA3 signals were detected, similar to the levels of RNA3 derived entirely from plasmid-based transcription in cells lacking 1a (Fig. 10A). In cells expressing Class II 1a mutants, 2a^Pol^ and RNA3, positive-strand RNA3 accumulated to levels intermediate between those in cells with and without wt 1a (Fig. 10A), consistent with the ability of class II 1a mutants to mediate RNA3 recruitment to a membrane-protected state (Fig. 9). However, positive-strand RNA4 and negative-strand strand RNA3, which are only synthesized as products of viral RNA replication, were undetectable in cells expressing any of the Class I 1a mutants, and reached only 5–10% of wt levels in cells expressing most Class II 1a mutants (Fig. 10A). The only exception was Class II mutant 1aT397A, which weakly stimulated 2a^Pol^ accumulation (Fig. 8C) and retained ∼25% of wt 1a replication levels (Fig. 10A). Thus, BMV RNA replication was severely inhibited by the helix A mutations in both classes.

To compare the replication competence of the 1a helix A mutants in yeast to that in BMV's natural plant host, 7-day old leaves of barley plants were inoculated with *in vitro* transcribed wt or mutant RNA1 transcripts and equal amounts of RNA2 and RNA3 transcripts. Seven to nine days post inoculation with wt BMV RNAs, even leaves that were not inoculated but rather depended on systemic viral spread for infection contained abundant levels of RNA1, 2, 3 and RNA4 (Fig. 10B, lane 1). However, none of the RNA 1 mutants, including 1aT397A, supported detectable systemic infection (Fig. 10B). Thus, 1a mutations in helix A that abolish or severely inhibit BMV RNA replication in yeast also render the virus severely replication-deficient in its natural host.

## Discussion

Positive-strand RNA virus RNA replication occurs exclusively on intracellular membranes. Thus, the interactions by which viral replication proteins target specific membranes, recruit other viral proteins and viral RNA templates, and reorganize their target membranes to accommodate active RNA replication compartments are crucial to understanding replication complex assembly and function [12],[23]. In the case of BMV, the multifunctional replication protein 1a directs replication complex targeting and ER membrane-association, in addition to providing all viral enzymatic functions for RNA replication other than polymerase activity. Previously, we mapped the major 1a ER membrane association-mediating sequences between aa 368 and 478 (region E), and membrane association enhancing sequences in upstream region D [24]. Additional contributions to 1a membrane association were mapped to the 158 N-terminal amino acids of 1a (region A&B) [24]. However, we found that region E was sufficient for ER targeting, whereas the auxiliary sequences in region A, B, and D were not. Here, we have used genetic, biochemical and NMR analyses to identify a small amphipathic α-helix within BMV 1a region E, helix A, that is not only critically involved in 1a-induced membrane association and rearrangement, but also in 1a-mediated recruitment of viral RNA templates and RNA polymerase, and subsequent assembly and function of active replication complexes.

### Roles of helix A in 1a-membrane interaction and 1a function

**Figure 11:**
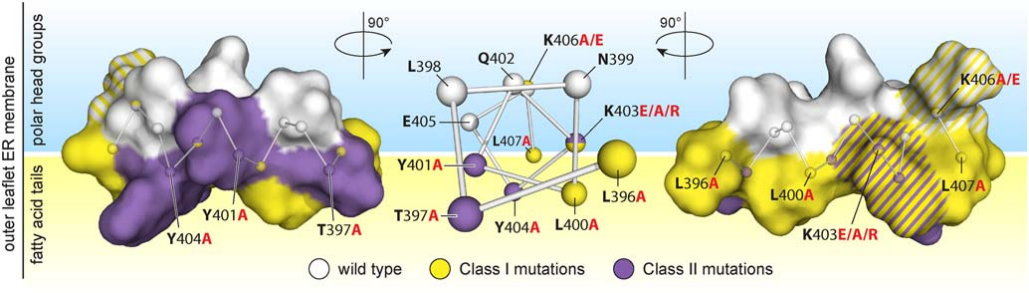
Class I and Class II mutations cluster on opposite sides of helix A. Center: “ball & stick” representation of the NMR-based membrane topology of the core 12 amino acids viewed along the N- to C-terminal axis of the helix. Positions of Class I and Class II mutations (with relevant mutant residues shown in red type) are mapped in yellow and purple, respectively. Left & Right: left side and right side views of the NMR-based, three-dimensional structure of helix A with the same coloring scheme. Note that positions K403 and K406 (hatched coloring) represent intermediate cases: while mutation K403E has a Class I phenotype and is on the same face as the other Class I mutations (right side), K403 is also immediately adjacent to Class II mutation Y404A, and mutation K403R has a class II phenotype. K406E has a weak Class I phenotype, while K406A and K406R have a wt 1a phenotype.

NMR structure analyses showed that at a minimum, the core twelve amino acids of helix A are in an α-helical configuration (Fig. 3). Mutational analyses (Fig. 4 and Fig. 5) and SDS micelle-based NMR and DSA-16 contact data (Fig. 3C and 3D and Fig. 11) show that the primary membrane association function of helix A resides in a hydrophobic face comprised primarily of three leucines at aa positions 396, 400, and 407. These leucines show significant conservation among sequenced bromoviruses (Fig. 1A), and mutation of these leucines to alanines, in effect removing their side chains, greatly diminished helix A- and full-length 1a-mediated membrane association (Fig. 4 and Fig. 5). Insertions of alanines immediately adjacent to the leucines, which disrupts their correct spacing and the amphipathic characteristics of helix A, likewise reduces 1a membrane association efficiency to that of complete helix A deletion mutants (Fig. 5). When lysines at positions 403 and 406, which can potentially interact with the negatively charged polar head groups of the lipid bilayer, were mutated to glutamic acids to change positive charge to negative charge, membrane association was also affected, but to a lesser extent (Fig. 4 and Fig. 5).

Immediately adjacent to the triple leucine hydrophobic face of helix A, T397, Y401, and Y404 form a polar and uncharged side (Fig. 3C, bottom view and 11). Polar residues Y and T are common targets for phosphorylation, which would add negative charge. However, computer-assisted predictions [30] do not support the likelihood of phosphorylation at these residues, due to lack of flanking phosphorylation site consensus sequences. Indeed, neither mutations T397D, Y401E and Y404E (intended to mimic phosphorylation), nor mutations T397R, Y401R and Y404R (that added positive charge) affected spherule formation, although they did render 1a defective in RNA replication (data not shown). Strikingly, however, alanine substitutions in this same T-Y-Y face of helix A revealed an entirely new 1a mutant phenotype. Unlike the L396/400/407 Class I mutants, these Class II mutants not only retained efficient ER membrane association, but dramatically increased the frequency of 1a-induced membrane invaginations 5- to 8-fold (Table 3).

Additional characterization further extended the opposing nature of the Class I and Class II 1a mutant phenotypes to the regulation of BMV 1a-mediated recruitment of 2a^Pol^ and viral RNA templates into the membrane-associated replication complex. Wildtype 1a directs cytosolic 2a^Pol^ to ER membranes via interaction of its C-terminal sequences with the N-terminal sequences of 2a^Pol^ [19], simultaneously stimulating 2a^Pol^ accumulation by ∼2-fold (Fig. 8A). Remarkably, Class I 1a mutations, which significantly inhibit 1a membrane affinity and abolish the capability to rearrange membranes, nearly doubled the ability of 1a to stimulate 2a^Pol^ accumulation (Fig. 8B). These 1a mutants decreased 1a membrane affinity to about 50% and, although interacting more efficiently with 2a^Pol^, they did not recruit 2a^Pol^ to the typical perinuclear ER location. Instead, co-expressing these Class I 1a mutants with 2a^Pol^ induced both to concentrate into large cytoplasmic clusters (Fig. 8D). In sharp contrast, stimulation of 2a^Pol^ accumulation by 1a was completely abolished when co-expressed with the Class II 1a mutants that induced dramatically more abundant spherules than wt 1a (Fig. 7).

Analysis of the Class I and II mutants also showed that RNA3 recruitment and protection by 1a, unlike 2a^Pol^ recruitment, strongly correlated with 1a-induced membrane invagination. Class I 1a mutants that did not induce ER membrane invaginations failed to mediate significant recruitment of template RNA3, while Class II 1a mutants that form hyper-abundant spherules enhance RNA3 accumulation to even higher levels than wt 1a (Fig. 9 and Table 3). Along with prior results [13], this implies that the membrane-associated, nuclease-resistant state associated with RNA3 recruitment (Fig. 9C) represents the spherule interior.

### Pathways of RNA replication complex assembly

**Figure 12:**
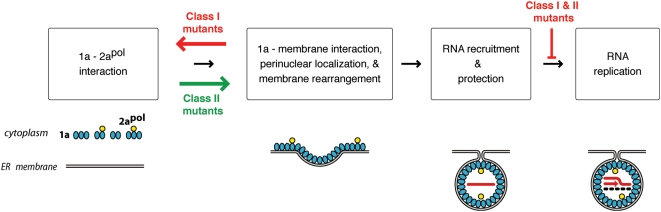
Sequential order of BMV RNA replication complex assembly steps. The order of replication complex assembly steps shown is as inferred from the effects of Class I and Class II mutations in helix A on complex assembly and other data (see main text for further details). The black arrows show the inferred progression of replication complex assembly for wt 1a. Consistent with the effects of Class I and II mutant phenotypes on membrane interaction and 2a^Pol^ recruitment (see below), and with the ability of 1a to recruit nascent 2a^Pol^ from translating, cytoplasmic polysomes [19], 1a and 2a^Pol^ interact in the cytoplasm prior to membrane association. For Class II mutants, subsequent 1a–membrane association and 1a-induced membrane rearrangement is correlated with inhibition of 1a-2a^Pol^ interaction. The effects of mutations in the C-proximal 1a NTPase/helicase domain imply that 1a-mediated recruitment of viral RNA templates to the membrane-associated, protected state required for replication occurs after 1a-induced membrane rearrangement [21], as shown. Red and green arrows show the opposite shifts in assembly equilibrium induced by Class I and Class II mutations. Class I 1a mutants have lost helix A–mediated ER membrane association and all capability to invaginate or otherwise modify membranes, but retain efficient interaction with 2a^Pol^. In contrast, Class II 1a mutants retain efficient ER membrane association and show greatly increased levels of membrane invagination and RNA template recruitment, but have decreased interaction with 2a^Pol^.

The Class I and II 1a mutant phenotypes reveal significant insights into the pathways by which BMV RNA replication complexes assemble (Fig. 12). Immunogold electron microscopy and stoichiometric calculations of the various viral components in wt BMV replication complexes indicate that each spherule replication complex contains ∼200–400 BMV 1a molecules [13]. Calculations of spherule surface area and the predicted size of the 1a protein, 1a's strong affinity for the cytoplasmic face of the ER membrane [24], 1a self-interaction [29], and other results all imply that 1a forms an inner shell inside the spherules, explaining the formation and maintenance of these high-energy membrane deformations [12],[13]. Similar conclusions, based on electron microscope tomography and multiple other approaches, recently emerged for the role of transmembrane viral replication protein A in spherule RNA replication complexes formed by flock house nodavirus on mitochondrial membranes [23]. The observation that the Class II cluster of helix A mutations alters the size of the induced membrane spherules (Table 3) suggests that this part of helix A affects 1a-membrane and/or 1a-1a self-interactions that determine the diameter of the inner protein shell. Such altered interactions, together with the ∼3-fold reduced volume of Class II spherules, explain how Class II 1a mutants produce significantly more spherules than wt 1a (Table 3) from a similar or only slightly increased number of 1a proteins (Fig. 3C).

Since 1a mutants in the triple-leucine Class I cluster fail to induce ER invaginations and have reduced membrane association, these mutant 1a proteins must remain exposed at the cytoplasmic surface of the ER membrane or dissociate entirely from the membrane (Fig. 12). Such Class I 1a mutants were over twice as effective as wt 1a at interacting with, stabilizing and recruiting 2a^Pol^ to membranes (Fig. 8 and Table 3). This implies that 1a-2a^Pol^ interactions occur most efficiently and perhaps exclusively prior to spherule formation (Fig. 12). Consistent with these findings, Class II mutants, which are hyper-active in spherule formation, were markedly defective in 2a^Pol^ stabilization (Fig. 8 and Table 3). These results suggest that 1a-2a^Pol^ interaction and 1a-induced membrane invagination are sequential and perhaps antagonistic functions. Spherule formation might interfere with 1a-2a^Pol^ interaction by sequestering 1a in the spherule interior, by inducing conformational changes in 1a, or both. In either case, such interference could help to explain how spherules regulate accumulation of 2a^Pol^ to catalytic amounts of only one 2a^Pol^ for every 20 1a molecules [13]. Since Class I mutants with reduced membrane association enhanced 1a-2a^Pol^ interaction and nascent 2a^Pol^ can be efficiently recruited by 1a from cytosolic, translating polysomes [31], 1a-2a^Pol^ interaction may preferentially occur prior to 1a-membrane association.

In contrast to their 2a^Pol^ recruitment phenotypes, the Class I and Class II 1a mutant phenotypes as noted above showed strong correlation between spherule formation and recruiting and protecting RNA3 templates (Fig. 9 and Table 3), which likely extends to the mechanistically very similar recruitment of RNA1 and RNA2 templates [20],[32],[33]. This implies that RNA template recruitment is either closely linked to or subsequent to spherule formation ([21] and Fig. 12). The resulting ordered progression of 2a^Pol^ recruitment, replication complex assembly and RNA template recruitment seems tailored to satisfy the virus's crucial survival need to effectively use the limited number of viral genomic RNAs - potentially one - present during early phases of infection. It also is consistent with the dual function of the viral RNA genome to serve as a template for replication only after it has been translated and sufficient amounts of viral replication proteins have accumulated.

In summary, we find that helix A has crucial roles in directing and/or regulating multiple essential 1a functions in RNA replication complex assembly and function, including binding to membranes, inducing membrane curvature, and interacting with itself, 2a^Pol^ and viral RNA templates. In addition, the fact that RNA replication was abolished or severely inhibited by all Class II mutations (Fig. 10), which preserved membrane interaction, invagination and RNA recruitment, suggests that helix A may affect one or more additional 1a functions required for RNA synthesis, such as the enzymatic functions of the 1a RNA capping or NTPase/helicase domains (Fig. 1). Similar to the central role of helix A in 1a, amphipathic α-helices are also essential for the peripheral membrane association and function of some other positive-strand RNA virus replication factors, such as the *Flaviviridae* NS5A membrane anchor [34] Semliki Forest virus nsP1 RNA capping protein [4], and picornavirus 2C protein [6]. As with the possible role of helix A in modulating 1a enzymatic activities, the RNA capping activity of nsP1 is dependent on membrane association by its short amphipathic helix [35]. Moreover, like 1a, picornavirus 2C not only associates with membranes through an amphipathic helix, but induces membrane rearrangements, has NTPase activity and conserved helicase motifs required for RNA replication, and is implicated in amphipathic helix-modulated interactions with other viral RNA replication proteins [6]. Such emerging commonalities suggest that the membrane interaction and function of such amphipathic helices may embody common principles extending across important virus groups.

## Materials and Methods

### Yeast and plasmids

Yeast strain YPH500 and culture conditions were as described previously [8]. BMV 1a and mutant derivatives and 2a^Pol^ were expressed under control of the *GAL1* promoter, using pB1YT3 [16] or derivatives and pB2YT5 [36], respectively. BMV RNA3 was expressed from pB3MS82, a *GAL1* promoter expression plasmid of an RNA3 derivative with a four-nucleotide insertion in the coat protein gene has, abolishing expression of the coat protein [36]. The Sec63-GFP fusion protein was expressed from plasmid pWSECG, a derivative of pJK59 (gift from P. Silver, Department of Biological Chemistry and Molecular Pharmacology, Harvard University). The yeast-enhanced version of GFP and GFP-2a^Pol^ were expressed from pGFP and pGFP-2a^Pol^, respectively, both based on pB2YT5 [19].

### Membrane flotation assay

Ten OD600 units of yeast cells grown to mid-logarithmic phase were spheroplasted [37] and resuspended in 350 µl buffer TNT (50 mM Tris-HCl [pH 7.4], 150 mM NaCl, 5 mM EDTA, 5 mM benzamidine, 1 mM PMSF, and 10 µg/ml each aprotinin, leupeptin, and pepstatin A). Spheroplasts were lysed via 25 passes through a 22 gauge, 4 cm long needle. Total lysates were centrifuged for 5 minutes at 4°C at 500×g to remove cell debris, and 250 µl of supernatants were mixed with 500 µl of 60% Optiprep (Axis-Shield, Oslo, Norway). Density gradient centrifugation was performed for 5 hours at 55,000 rpm in a Beckman TLS55 rotor using 600 µl of each sample overlaid by 1.4 ml of 30% Optiprep and 100 µl of lysis buffer [21] After centrifugation, 6 fractions were collected from top to bottom of the gradient. For protein detection, samples were boiled in SDS loading buffer prior to SDS-PAGE and western blotting. For RNA analysis, RNA was isolated and prepared by the hot phenol method [38], and northern blotting was performed as described previously [21].

### Cell fractionation

Spheroplasts were lysed in 100 µl lysis buffer (50 mM Tris-Cl pH 8.0, 2.5 mM EDTA, 1 mM PMSF, 5 µg/ml pepstain, 10 µg/ml leupeptin, 10 µg/ml aprotinin, 10 mM benzamidine) and centrifuged 5 min at 4°C at 2000×g to yield pellet and supernatant fractions. For RNase treatment, 1 U of micrococcal nuclease was added to 100 µl of supernatant and pellet fractions, incubated at 30°C for 15 minutes and inactivated by addition of 2 µl of 0.5 M EGTA (pH 8.0). RNA was isolated and prepared by the hot phenol method [38], and northern blotting was performed as previously described [21].

### Plant inoculation, RNA isolation, and northern blot analyses

BMV RNA1 or its mutants, RNA2, and RNA3 were *in vitro* transcribed and capped (Ambion, Austin, TX) from EcoRI-linearized plasmid pB1TP3 or its derivatives, pB2TP5 and pB3TP8, respectively [39]. Seven-day-old barley leaves were inoculated with the resulting in vitro transcripts [40] and viral RNA was isolated seven to nine days post inoculation using a Qiagen RNeasy Mini kit. Northern blotting was performed as previously described [21].

### Peptide synthesis

The helix A peptide, representing amino acids 392–409 of BMV 1a (GenBank accession number ABF83485), was synthesized on an Applied Biosystems 432A synthesizer using standard Fmoc chemistry with HBTU/HoBT coupling [41]. Except for phenylalanine, all Fmoc-^15^N, (U)-^13^C labeled amino acids were purchased from Cambridge Isotope Laboratories. Labeled Fmoc-phenylalanine was purchased from Isotech. Wang resin was loaded with Fmoc-^15^N, (U)-^13^C-alanine using N, N-Diisopropylcarbodiimide and 4-diththylaminopyridine. The synthesis was carried out at a scale of 12.5 micromoles with a 3-fold excess of each amino acid. Coupling times for the first three and final 5 couplings were fixed at one hour each. The remaining 6 couplings were programmed as extended couplings. The cleaved and deprotected peptide was purified by HPLC using a C18 Vydac column (250×10 mm). Mass confirmation was done using a Bruker Biflex III MALDI-TOF.

### NMR analysis

NMR spectra were collected from a solution of 400 mM peptide in 100 mM SDS and 5% ^2^H_2_O using a Varian VNMRS 600 MHz spectrometer equipped with a 5 mm cryogenic triple resonance probe. DSA (4,4-dimethyl-4-silapentane-1-ammonium trifluoroacetate) and deuterated SDS were purchased from Aldrich. The 3D data were collected using HIFI, a rapid methodology for collection of multidimensional NMR spectra [42]. Spectra collected for assignments were: ^1^H{^15^N}HSQC, HNCO, CBCA(CO)NH, HNCACB, HNCA, HN(CO)CA, HN(CA)CO, HN(CA)CB. All experiments were standard Varian Biopack pulse sequences modified for the HIFI method [42].These sequences are available from the National Magnetic Resonance Facility at Madison. Automatically generated peak lists from HIFI were used as input to the automated assignment package suite (PISTACHIO [43], LACS [44], and PECAN [45]. 3D ^15^N-edited ^1^H-^1^H NOESY and 3D ^13^C-edited ^1^H-^1^H NOESY spectra were used as input for the ATNOS/CANDID/CYANA suite of programs [46],[47],[48]. The Protein Structure Validation Software suite of programs was used to assess the quality of the computed structure [49]. Images were rendered using PyMOL molecular graphics software (DeLano Scientific LCC http://www.pymol.org). The NMR and structural data described have been deposited in BioMagResBank (http://www.bmrb.wisc.edu) under BMRB accession number 20027.

### Immunofluorescence and confocal microscopy

Confocal microscopy was as described [21],[27]. Briefly, yeast cells co-expressing either wt 1a or 1a mutants and Sec63-GFP or GFP-2a^Pol^ were fixed with 5% formaldehyde, spheroplasted with lyticase, and permeabilized with 0.1% Triton X-100. Spheroplasts were then stained by using rabbit anti-1a serum, followed by Texas red-conjugated donkey anti-rabbit antibodies. Fluorescent images were acquired with a Bio-Rad 1042 double-channel confocal microscope system.

### Electron microscopy

Samples were prepared for electron microscopy as described [13]. In brief, yeast cells were fixed for 1 hr with 2% glutaraldehyde and 4% paraformaldehyde, washed, and post-fixed for 1 hr with 1% OsO_4_ and 1% uranyl acetate. Cells then were dehydrated via a series of step-wise increasing ethanol concentrations ranging from 50% to 100%, and infiltrated and embedded with Spurrs resin. Samples were sectioned and placed on nickel grids, washed, incubated in 15 min in 2% glutaraldehyde, poststained with 8% uranyl acetate and Reynold's lead citrate, and viewed with a Philips CM120 microscope.
